# Phytochemical Composition and In Vitro Antimicrobial Activity of Essential Oils from the *Lamiaceae* Family against *Streptococcus agalactiae* and *Candida albicans* Biofilms

**DOI:** 10.3390/antibiotics9090592

**Published:** 2020-09-10

**Authors:** Ramona Iseppi, Roberta Tardugno, Virginia Brighenti, Stefania Benvenuti, Carla Sabia, Federica Pellati, Patrizia Messi

**Affiliations:** Department of Life Sciences, University of Modena and Reggio Emilia, Via G. Campi 103/287, 41125 Modena, Italy; roberta.tardugno@gmail.com (R.T.); virginia.brighenti@unimore.it (V.B.); stefania.benvenuti@unimore.it (S.B.); carla.sabia@unimore.it (C.S.); patrizia.messi@unimore.it (P.M.)

**Keywords:** essential oil, *Lamiaceae*, *Lavandula x intermedia*, *Mentha arvensis*, *Streptococcus agalactiae*, *Candida albicans*, antibiotics, synergic associations, anti-biofilm activity

## Abstract

The antimicrobial activity of different essential oils (EOs) from the *Lamiaceae* family was evaluated on *Streptococcus agalactiae*, *Candida albicans*, and lactobacilli. *S. agalactiae* is the main cause of severe neonatal infections, such as sepsis, meningitis, and pneumonia. *C. albicans* is a primary causative agent of vulvovaginal candidiasis, a multifactorial infectious disease of the lower female reproductive tract. Lactobacilli represent the dominant bacterial species of the vaginal flora and constitute the natural defense against pathogens. On the basis of the preliminary results, the attention was focused on the EOs from *Lavandula x intermedia* Emeric ex Loisel. and *Mentha arvensis* L. By using gas ghromatography (GS) retention data and mass spectra, it was possible to identify more than 90% of the total composition of the EO samples. The minimal inhibitory concentration (MIC) and anti-biofilm activity of the two EOs were determined against all isolated strains, using the EOs by themselves or in combination with each other and with drugs (erythromycin and fluconazole). The results showed a good antimicrobial and anti-biofilm activity of both EOs and a synergistic effect, leading to the best results against all the strains, resulted using the combinations EOs/EOs and antimicrobials/EOs.

## 1. Introduction 

The vaginal flora is strongly related to women health. The vaginal ecosystem is typically dominated by different *Lactobacillus* species, mainly *Lactobacillus crispatus*, *Lactobacillus gasseri*, *Lactobacillus iners*, and *Lactobacillus jensenii*. They play a protective role by a combination of various mechanisms, i.e., by maintaining a low pH (<4.5), by a specific adhesion to the vaginal tissue, and by the production of inhibitory substances (e.g., organic acids, hydrogen peroxide, bacteriocins), thus reducing the development of most pathogenic microorganisms [1,2]. The lactobacilli reduction leads to an alteration of vaginal flora and, consequently, favors the pathogenic bacterial growth with health outcomes. *Streptococcus agalactiae*, also known as the Group B *Streptococcus* (GBS), is a Gram-positive, facultative anaerobe and an opportunistic pathogen for pregnant women, newborns, and elderly persons. GBS colonizes the female genital tract (50% of the female population), the gastrointestinal tract and the urethra of both men and women [3]. Between 10 and 30% of pregnant women are intermittently or persistently colonized by GBS and they are at risk of transmitting this pathogen to their newborn infant in the perinatal period [4,5]. GBS can be life-threatening, when it is vertically transmitted through the birth canal from a colonized mother to her newborn at birth, causing pneumonia, meningitis, and sepsis. 

The most accepted medical procedure for the detection of GBS in pregnant women is a maternal screening for clinical risk factors at delivery or rectovaginal colonization only at 35–37 weeks of gestation and, in the presence of this pathogen, a subsequent intrapartum antibiotic prophylaxis is prescribed [6]. With few exceptions, GBS remains fully susceptible to penicillin. However, about 10% of pregnant women are allergic to penicillin and an alternative to this antibiotic should be administered. Erythromycin and clindamycin are the most used treatments, but GBS has increased over time its resistance to these drugs [7].

*Candida albicans* is a normal constituent of human flora, and a common colonizer of mucosal membrane, skin, gastrointestinal tract, and vaginal mucosa. In more than 75% of cases, this species behaves as an opportunistic pathogen responsible for vulvovaginal candidiasis (VVC) [8,9,10,11]. The ability of *Candida* species to form biofilm is considered as a critical point in its pathogenesis, with an increased resistance to traditional antimycotic agents [9,12,13,14]. Fluconazole is the first-line antifungal agent used in clinical VVC infections [13]. However, variable sensitivity or resistance to this drug reduces the effectiveness of the treatment, thus inducing recurrent vaginal candidiasis [10,15,16,17,18,19,20,21]. 

This growing trend indicates that there is a crucial need for highly efficient antibacterial and antifungal alternative agents with few side effects. The vast structural diversity of natural compounds of plant origin provides a unique opportunity for discovering new bioactive molecules. In this ambit, essential oils (EOs) represent an alternative to conventional antimicrobial treatments, due to their broad-spectrum activity against microorganisms, mainly as a consequence of the alteration of the microbial membrane and cell wall, with resulting loss of cytoplasmic material. EOs biological activities are due to their complex chemical composition and presence of phenols, which are compounds of interest for the treatment of both bacterial and fungal infections [9,22,23,24,25,26,27]. 

In this work, different essential oils (EOs) belonging to the *Lamiaceae* family were evaluated for their antimicrobial activity against two *S. agalactiae* and two *C. albicans* strains isolated from vaginal swabs. Four *Lactobacillus* strains were also tested since, during women fertile age, they represent 90% of vaginal flora, and they play an important role in protecting the host from genital infections.

## 2. Results

### 2.1. Agar Disk Diffusion Assay 

As shown in Table S1, erythromycin and fluconazole displayed their activity against *Streptococcus agalactiae* and *Candida albicans* strains, respectively. These two antimicrobials used as the reference showed their activity also towards lactobacilli. By using the agar disk diffusion method and considering the zone of inhibition, it was also possible to verify the EOs inhibitory activity against all the microorganisms tested. Among the EOs used, *Lavandula x intermedia* Emeric and *Mentha arvensis* L. were the most active against all *C. albicans* strains, even if a less evident activity of *M. arvensis* EO towards the *C. albicans* 2 strain emerged. *L. x intermedia* showed also a good antibacterial activity against *S. agalactiae*, in particular, towards *S. agalactiae* ATCC 13813. *Satureja montana* and *Thymus vulgaris* EOs displayed good activity against *S. agalactiae*, while their activity towards *C. albicans* strains was lower than *L. x intermedia* and *M. arvensis* EOs. *M. arvensis* EO exhibited also antimicrobial activity against lactobacilli, if compared to *L. x intermedia* EO. *L. x intermedia* EO was selected for further chemical analysis, due to its excellent antimicrobial activity against *S. agalactiae* and *C. albicans* strains and its low activity towards lactobacilli. *M. arvensis* was chosen for its activity against candida, a microorganism found to be frequently resistant to fluconazole, the first-line antifungal agents used in clinical VVC infections, and often responsible for recurrent vaginal diseases.

### 2.2. Qualitative and Semi-Quantitative Analysis of EOs

On the basis of the preliminary results obtained by the agar disk diffusion assay, a phytochemical characterization of *L. x intermedia* and *M. arvensis* EOs was carried out by means of gas chromatography (GC). By using retention data, mass spectra, and data reported in the literature, it was possible to identify the analytes in all the EO samples (Table 1). More than 90% of the total composition for each EO was characterized.

*L. x intermedia* EO showed a composition rich in linalool (36.0%), linalyl acetate (27.3%). Other peculiar constituents of this hybrid were camphor (5.9%), 1,8-cineole (5.0%), and borneol (4.0%) [28,29]. *M. arvensis* EO main compounds included menthol (73.8%), menthone (7.8%), isomenthone (5.4%), limonene (3.6%), and menthyl acetate (2.1%). The two EOs composition was in accordance with the data reported in the literature [30].

### 2.3. Minimum Inhibitory Concentration (MIC) 

The minimum inhibitory concentrations (MICs) against all strains of both antimicrobials confirmed the results of the disk diffusion test (Table 2). As regards the EOs, the best antimicrobial activity was observed for *L. x intermedia* EO, with values ranging from 9 to 18 μg/mL against all *S. agalactiae* and *C. albicans* strains. *M. arvensis* EO, as already observed with the agar disk diffusion assay, resulted less active against all the pathogenic strains, with values ranging from 18 to 144 μg/mL. On the contrary, higher MIC values were obtained for all the *Lactobacillus* strains treated with *L. x intermedia* EO than with *M. arvensis* EO. 

### 2.4. Determination of the Fractional Inhibitory Concentration Index (FICI)

The fractional inhibitory concentration (FIC) index evaluation indicated the absence of antagonistic effects and showed several synergistic interactions, in particular, between the two EOs against *S. agalactiae* and *C. albicans* strains (Table S2). Furthermore, it is important to underline the synergistic effect of the two EOs with erythromycin and fluconazole, even if less evident for the combination *M. arvensis* EO/erythromycin. The synergic combination as this could allow a decrease in the concentration of antimicrobial necessary for the therapeutic treatment.

### 2.5. Time–Kill Studies

**Figure 1:**
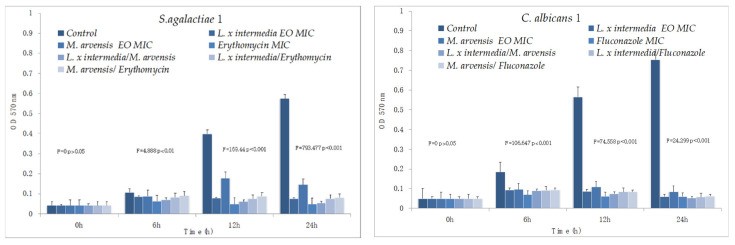

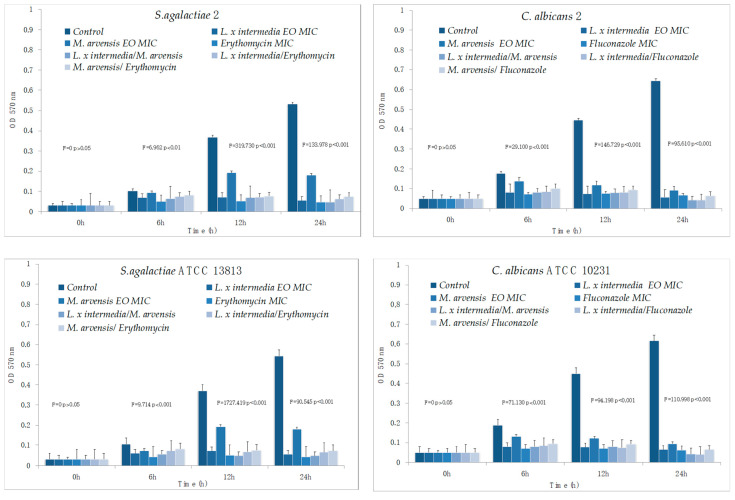
Time–kill studies of *L. x intermedia*, *M. arvensis* EOs, antimicrobials (erythromycin and fluconazole) and of different combinations (antimicrobial/EO and EO/EO) against *Streptococcus agalactiae* and *Candida albicans* strains. *p*-values of <0.05, *p* < 0.01, *p* < 0.001 and *p* < 0.0001 were considered significant by *t*-test and ANOVA.

**Figure 2:**
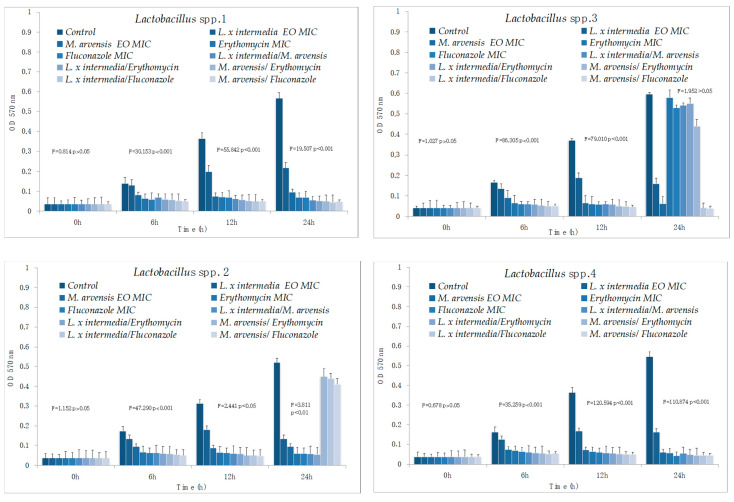
Time–kill studies of *L. x intermedia*, *M. arvensis* EOs, antimicrobials (erythromycin and fluconazole), and of different combinations (antimicrobial/EO and EO/EO) against *Lactobacillus* spp. strains. *p*-values of <0.05, *p* < 0.01, *p* < 0.001 and *p* < 0.0001 were considered significant by *t*-test and ANOVA.

The results of time–kill studies are shown in Figure 1 and Figure 2. Both EOs were active against all *S. agalactiae* and *C. albicans* strains, even if *L. x intermedia* EO was more effective than *M. arvensis* EO. A synergistic activity against *S. agalactiae* and *C. albicans* was observed with the combination antimicrobial/EO, and in particular when the two antimicrobials were used in association with *L. x intermedia* EO. The synergistic activity also emerged with the combination EO/EO against all *S. agalactiae* and *C. albicans* test strains. Lastly, *M. arvensis* EO reduced the presence of lactobacilli more than *L. x intermedia* EO. The anti-lactobacilli activity was detected with the synergic combination antibiotic/EO and EO/EO only for two *Lactobacillus* strains. Erythromycin and fluconazole activity against all tested strains confirmed the sensitivity patterns.

### 2.6. Anti-Biofilm Activity Determination

**Figure 3:**
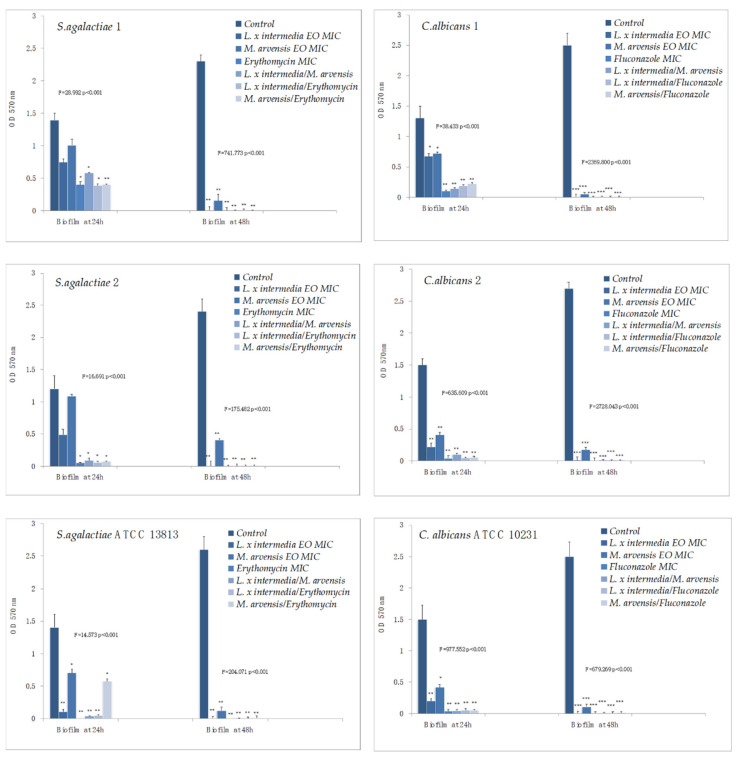
Anti-biofilm activity of *L. x intermedia*, *M. arvensis* EOs, antimicrobials (erythromycin and fluconazole), and of different combinations (antimicrobial/EO and EO/EO) against *S. agalactiae* and *C. albicans* strains. Results were expressed in optical density (OD) 570 nm as the arithmetic mean of the three determinations. *p*-values of <0.05 (*), *p* < 0.01 (**), *p* < 0.001 (***) were considered significant by *t*-test and ANOVA.

Erythromycin and all the combinations (EO/EO and antibiotic/EO) were effective against the mature biofilm of *S. agalactiae* strains. The synergistic effect of the EOs used in combination resulted more evident against the ’48 h old’ mature biofilm and, for the single EO, with a significant difference with the control (range of *p*-value from 0.004112 to 0.001285). Concerning *C. albicans* strains, *C. albicans* 2 and *C. albicans* ATCC 10231 in particular, fluconazole and the synergistic combinations (EO/EO and antifungal/EO) showed an excellent anti-biofilm activity. In the whole, *L. x intermedia* EO exhibited a better activity than *M. arvensis* EO against both the ‘24 h and 48 h old’ mature biofilm, proving to be the most effective product capable of counteracting its development (Figure 3). 

**Figure 4:**
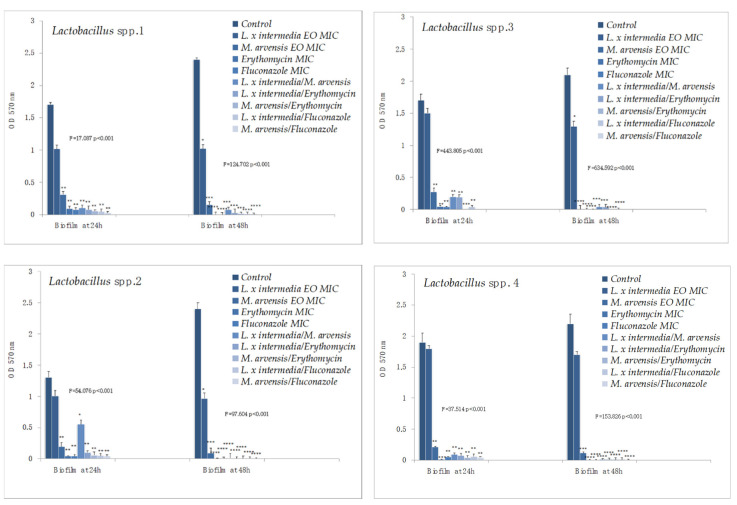
Anti-biofilm activity of *L. x intermedia*, *M. arvensis* EOs, antimicrobials (erythromycin and fluconazole), and of different combinations (antimicrobial/EO and EO/EO) against *Lactobacillus* spp. strains. Results were expressed in OD 570 nm as the arithmetic mean of the three determinations. *p*-values of <0.05 (*), *p* < 0.01 (**), *p* < 0.001 (***) and *p* < 0.0001 (****) were considered significant by *t*-test and ANOVA.

Lastly, *L. x intermedia* EO did not exhibit a remarkable activity towards the ‘24 h and 48 h old’ mature biofilm produced by *Lactobacillus* strains. Conversely, *M. arvensis* EO, fluconazole, and all the associations showed a good anti-biofilm activity against ‘24 h old’ mature biofilm of the same (*p*-value ranging from 0.0324 to 0.0041) (Figure 4). 

### 2.7. Quantification of EOs Activity on Mature Biofilm by Fluorescence Assay Study

We investigated biofilm changes following single EOs and their combination treatments. The ’24 h old’ mature biofilm was treated either with the EOs alone or in combination (EO/EO and antimicrobial/EO) and then stained with CFDA and PI to define a numerical quantification of live and dead cells.

**Figure 5:**
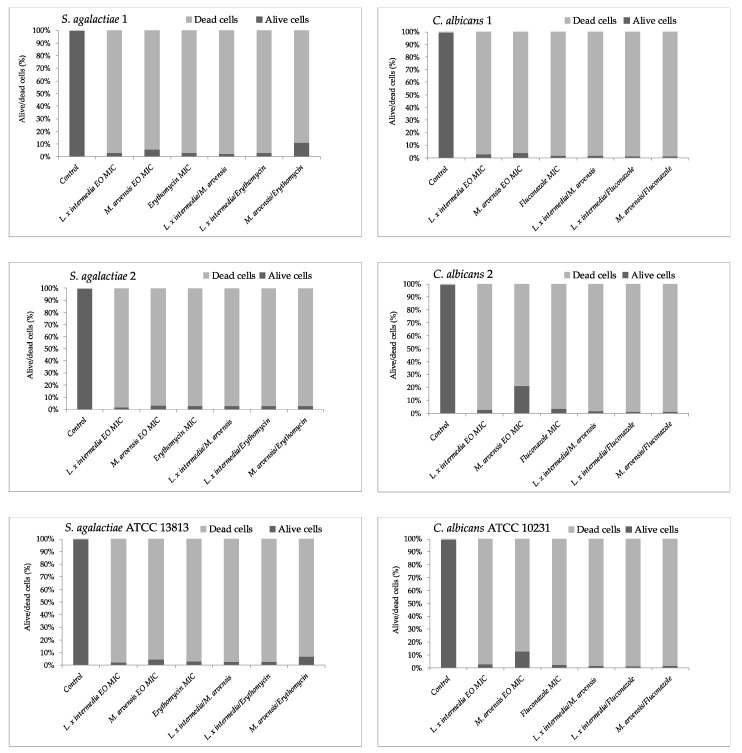
Quantification of alive/dead cells in the biofilm. Relative fluorescence units (RFU) mean of alive and dead cells into *S. agalactiae* and *C. albicans* biofilm, treated with *L. x intermedia* and *M. arvensis* EOs, antimicrobials (erythromycin and fluconazole), and with different combinations (antimicrobial/EO and EO/EO). The data shown are representative of three determinations. Statistically significant difference between alive and dead cells (*p*-values < 0.05) was detected by Qui-square test.

**Figure 6:**
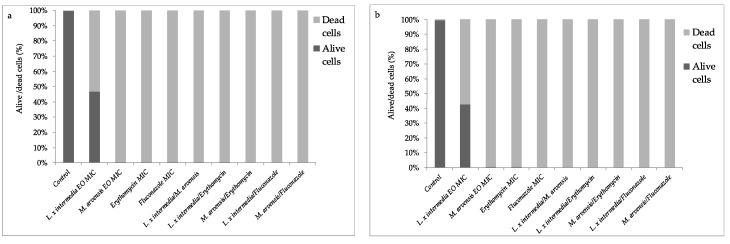

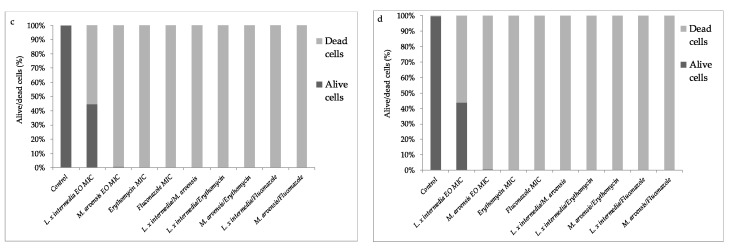
Quantification of alive/dead cells in the biofilm. Relative fluorescence units (RFU) mean of alive and dead cells into *Lactobacillus* spp. 1, *Lactobacillus* spp. 2, *Lactobacillus* spp. 3, and *Lactobacillus* spp. 4 ((**a**–**d**), respectively) biofilm, treated with *L. x intermedia* and *M. arvensis* EOs, antimicrobials (erythromycin and fluconazole) and with different combinations (antimicrobial/EO and EO/EO). The data shown are representative of three determinations. Statistically significant difference between alive and dead cells (*p*-values < 0.05) was detected by Qui-square test.

The EOs from *L. x intermedia* and *M. arvensis*, as well as the combinations (EO/EO and antimicrobial/EO) strongly reduced the number of viable cells of all tested strains (Figure 5 and Figure 6). *L. x intermedia* EO was more active against *S. agalactiae* and *C. albicans* strains in comparison with *M. arvensis* EO. On the other hand, *L. x intermedia* EO was less effective against all tested lactobacilli, as previously observed.

### 2.8. Effectiveness of EOs Activity on Mature Biofilm by Fluorescence Microscopy Study 

**Figure 7:**
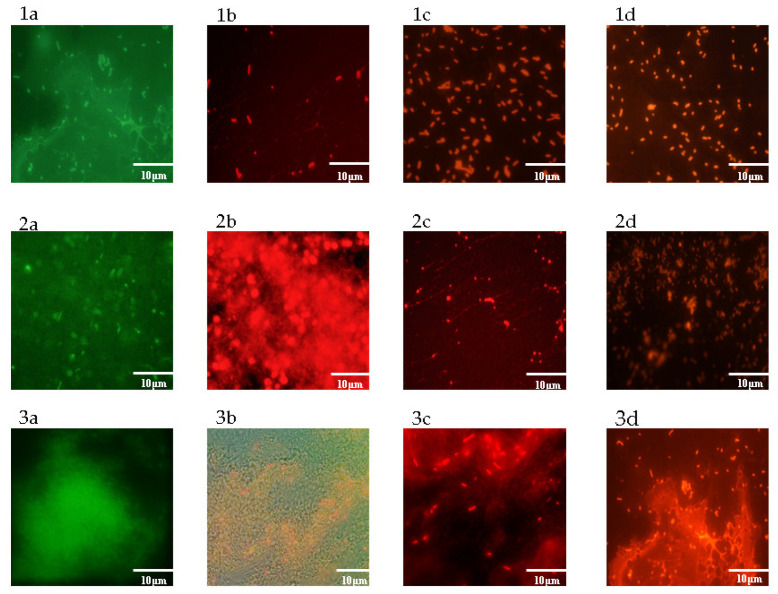
Images of light microscopy obtained by using “live/dead cells stain kit” for: *S. agalactiae* (**1a**), *C. albicans* (**2a**), and *Lactobacillus* spp. (**3a**) mature biofilm; *S. agalactiae* (**1b**), *C. albicans* (**2b**) and *Lactobacillus* spp. (**3b**) mature biofilm treated with *L. x intermedia* EO; *S. agalactiae* (**1c**), *C. albicans* (**2c**) and *Lactobacillus* spp. (**3c**) mature biofilm treated with *M. arvensis* EO; *S. agalactiae* (**1d**), *C. albicans* (**2d**) and *Lactobacillus* spp. (**3d**) mature biofilm treated with synergistic combination EO/EO. Green fluorescence labels live cells, whereas red fluorescence labels dead cells. The scale bars indicate 10 μm.

The anti-biofilm activity was assessed against all tested strains, by using live/dead staining in association with the observation under a fluorescence microscope. After treatment with the EOs themselves and their combinations (EO/EO and antimicrobial/EO), an abundant red fluorescence indicating dead cells in the polymeric matrix of *S. agalactiae*, *C. albicans*, and lactobacilli biofilm was observed (Figure 7). With regard to these last strains, the presence of viable lactobacilli in the sample treated with *L. x intermedia* EO once again indicates the reduced impact of this EO on the natural protective vaginal microflora.

## 3. Discussion

EOs are widely used as a possible alternative therapy for their antimicrobial effects [31,32]. The screening of medical plants, and in particular of those rich in EOs, for antibacterial and antifungal activities is important to find new active antimicrobial compounds. Many studies on the antibacterial activity of *Lavandula* EOs have been based on *Lavandula angustifolia* Miller [29,33], even if other varieties, such *L. x intermedia*, have shown therapeutic potential [34]. *Lavandula* species have also demonstrated to be a weapon against antimicrobial resistant pathogens responsible for human infections [35,36,37,38]. Many studies have focused on the antibacterial effects of *Lavandula* volatile compounds, such as linalool, linalyl acetate, and terpinen-4-ol [39,40]. EOs from *Mentha* species have also been used as a folk medicine, due to their antibacterial and antifungal activities attributed to high levels of monoterpenes, including menthol and menthone [41,42,43]. *M. arvensis*, also known as wild mint or corn mint, is one species with a high content of menthol (80–95%) [44]. 

In this study, two EOs (i.e., *L. x intermedia* and *M. arvensis*) were selected from a panel of EOs belonging to the *Lamiaceae* family on the basis of their antimicrobial activity against *S. agalactiae* and *C. albicans*, vaginal colonizers, and opportunistic pathogens, respectively. *S. agalactiae* colonizes female genital tract and it is the main cause of neonatal diseases, being transmitted vertically from a colonized mother to her newborn at birth [3,4,5]. Candida vaginitis is a complicated disease with wide-reaching effects that are governed by fungal biology and host physiology and response [9]. 

The natural defense against infections in the vaginal tract includes microbial flora, such as lactobacilli, which are believed to interfere with pathogens [19]. Several clinical studies have highlighted an inverse correlation between the presence of *Lactobacillus* species and *S. agalactiae* in female vaginal tract [45,46]. Lactobacilli can be able to protect from *Candida* colonization and infection because of their capability to adhere and compete for vaginal mucosa adhesion sites [47].

In light of all the above, the chemical characterization as well as the antibacterial and antifungal activities of *L. x intermedia* and *M. arvensis* EOs against *S. agalactiae*, *C. albicans*, and *Lactobacillus* species were assessed. The results clearly indicated that these EOs, having a high content of linalool and linalyl acetate for *L. x intermedia* and menthol for *M. arvensis*, respectively, showed a good antimicrobial activity against the two vaginal pathogens, in planktonic and in biofilm forms, both individually and in association with each other and with drugs (erythromycin and fluconazole). As reported for other medical and aromatic plants belonging to the *Lamiaceae* family, the anti-biofilm activity of EOs may occur through the modulation of certain genes involved in adhesion, a feature essential for biofilm formation and pathogenesis of microorganisms [48].

Other authors have already described the antimicrobial activity of *Lavandula* and *Mentha* EOs against *Candida albicans* and *S. agalactiae* isolated from vaginal swabs [49]. In addition, Minooeianhaghighi et al. have demonstrated that *Lavandula* EO is able to inhibit *C. albicans*, also in association with other EOs. The antibacterial activity of *Lavandula* EO seems closely related to amount of 1,8-cineol [49]. The anti-candida activity of *Mentha* EO has been described by other authors [50]. Pietrella et al. have found an antifungal activity of *Mentha* EO against azole-resistant *C. albicans* strains [50]. The antibacterial activity of these EOs could be due to their ability to degrade membrane proteins and cell permeability. Regarding anti-biofilm formation, EOs are able to inhibit adhesion of bacterial cells at the first stage of biofilm formation, which might be due to their capability to inhibit the quorum sensing (QS) activity, that enables bacterial cells to have a multicellular behavior [51]. Further study of the anti-QS activity of EOs should be analyzed in detail.

The single EOs and their combinations exhibited antibacterial activity also towards lactobacilli. *L. x intermedia* EO was the less active against lactobacilli, thus allowing the recognized protective function of lactic flora on vaginal mucosa. Therefore, this natural product could be used in the clinical field for the control of the pathogens, respecting the survival of lactobacilli. Nevertheless, further studies are needed, especially on the activity and toxicity of the EO constituents, that can be also responsible for undesirable effects, such as irritation, blushes, phototoxicity, sensitizations, which are still limiting their medicinal use.

## 4. Materials and Methods 

### 4.1. Chemicals and Reagents

All reference standards used for GC analysis, chromatographic grade organic solvents, and reagents were purchased from Sigma-Aldrich (Milan, Italy). 

### 4.2. Essential Oils

Authentic EOs samples from *L. x intermedia*, *M. arvensis*, *Origanum vulgare* L., *Satureja montana* L., and *Thymus vulgaris* L., all obtained by hydrodistillation, were kindly provided by Alchimia Natura srl (Modena, Italy). The samples were stored at low temperature (+4 °C), protected from light and humidity, until required for chemical analysis.

### 4.3. Microbial Strains

All the strains were collected in June 2017 from the Provincial Laboratory of Clinical Microbiology ‘S. Agostino-Estense’ Hospital (Modena, Italy). Vaginal swabs of women aged between 25 to 40 were randomly selected. The *C. albicans* (*n* = 2, *C. albicans* IP01 and *C. albicans* IP02) and *S. agalactiae* (GBS) (*n* = 2, *S. agalactiae* IP03 and *S. agalactiae* IP04) strains were isolated on CHROMID agar (CHROMID^®^ Candida^®^ Candida, bioMérieux, Milan, Italy) and on group B *Streptococcus* differential agar (CHROMID^®^ Strepto B, bioMérieux, Milan, Italy), respectively. Four strains of lactobacilli (*Lactobacillus* spp. 1-IP04, *Lactobacillus* spp. 2-IP05, *Lactobacillus* spp. 3-IP06, and *Lactobacillus* spp. 4-IP07) were also isolated in Man-Rogosa Sharpe agar (MRS agar, bioMérieux, Milan, Italy). 

All the isolates were confirmed by matrix-assisted laser desorption ionization (MALDI) time-of-flight mass spectrometry (TOF/MS). *C. albicans* ATCC 10231 and *S. agalactiae* ATCC 13813 were included as positive controls. All strains were maintained in the same media containing 20% (*w*/*v*) glycerol at −80 °C until use.

### 4.4. Agar Disk Diffusion Assay 

The preliminary determination of the effectiveness of the above mentioned EOs against all the microorganisms was carried out by using the agar disk diffusion assay, according to the standard procedure of the Clinical and Laboratory Standards Institute [52]. Sterile disks of 6 mm in diameter, containing 10 μL of each EO, were placed on Tryptic Soy Agar (TSA, Oxoid) plates, previously seeded with 100 μL of 10^6^ CFU/mL of cell suspensions. Erythromycin (15 μg) and fluconazole (25 μg) discs were used as the positive controls. After incubation at 37 °C for 24 h, in anaerobic condition for GSB and lactobacilli strains, the antagonistic activity of the EOs was quantified by a clear zone of inhibition in the indicator lawn around the disks and the diameters in millimeters of these zones were measured [53].

### 4.5. GC Analysis

GC-MS analyses were performed on a 7890A gas chromatograph coupled with a 5975C network mass spectrometer (Agilent Technologies, Germany). Compounds were separated on an Agilent Technologies HP-5 MS cross-linked poly-5% diphenyl–95% dimethyl polysiloxane (30 m × 0.25 mm i.d., 0.25 μm film thickness) capillary column. The column temperature was initially set at 45 °C, then increased at a rate of 2 °C/min up to 100 °C, then raised to 250 °C at a rate of 5 °C/min and finally held for 5 min. The injection volume was 0.1 μL, with a split ratio 1:20. Helium was used as the carrier gas, at a flow rate of 0.7 mL/min. The injector, transfer line, and ion-source temperature were 250, 280, and 230 °C, respectively. MS detection was performed with electron ionization (EI) at 70 eV, operating in the full-scan acquisition mode in the *m*/*z* range 40–400. EOs were diluted 1:20 (*v*/*v*) with *n*-hexane before GC-MS analysis. 

GC analyses with flame ionization detector (FID) were carried out on a 7820 A from Technologies. Compounds were separated on an Agilent Technologies HP-5 cross-linked poly-5% diphenyl–95% dimethyl polysiloxane (30 m × 0.32 mm i.d., 0.25 μm film thickness) capillary column. The temperature program was the same as described above. The injection volume was 0.1 μL in the split mode 1:20. Helium was used as the carrier gas at a flow rate of 1.0 mL/min. The injector and detector temperature were set at 250 and 300 °C, respectively. The EOs and the reference standards were diluted to 1:20 (*v*/*v*) with n-hexane before GC-FID analysis. The analyses were performed in triplicate for each sample.

### 4.6. Qualitative and Semi-Quantitative Analysis

Compounds were identified by comparing the retention times of the chromatographic peaks with those of authentic reference standards run under the same conditions and by comparing the *LRI*s relative to C_8_-C_40_ *n*-alkanes obtained on the HP-5 column under the above-mentioned conditions with the literature [54]. Peak enrichment by co-injection with authentic reference compounds was also carried out. Comparison of the MS-fragmentation pattern of the target analytes with those of pure components was performed. A mass-spectrum database search was carried out by using the National Institute of Standards and Technology (NIST, Gaithersburg, MD, USA) mass-spectral database (version 2.0d, 2005). 

Semi-quantification was calculated as the relative percentage amount of each analyte; in particular, the values were expressed as the percentage peak area relative to the total composition of each EO obtained by GC-FID analysis. 

### 4.7. Minimum Inhibitory Concentration (MIC)

The MIC values of *L. x intermedia* and *M. arvensis* EOs were determined against all microorganisms by means of a microwell dilution method. The test was performed in sterile 96-well microplates by dispensing into each well 95 µL of nutrient broth and 5 µL of cell suspensions, to final inoculums concentrations of 10^6^ CFU/mL. Then, 100 µL of EO serial dilutions were added to obtain concentrations ranging from 512 to 0.25 μg/mL [55]. The last well, containing 195 μL of nutrient broth and 5 μL of the test strains without EOs, was used as the negative control. The antibiotics erythromycin and fluconazole, diluted in nutrient broth with strains added, were used as the positive control. The plates were incubated at 37 °C for 24 h, mixed on a plate shaker at 300 rpm for 20 s, and the MIC was defined as the lowest concentration of the EOs that inhibited visible growth of the tested microorganisms after measuring the optical density (OD) at 570 nm, using a microtiter plate reader. All the experiments were repeated three times.

### 4.8. Determination of the Fractional Inhibitory Concentration Index (FICI)

The checkerboard method was carried out in nutrient broth by using the microdilution method to obtain the FICI for the combined application of *L. x intermedia* and *M. arvensis* EOs and the combined effects of EOs and antimicrobials against all test strains. FICI was calculated as follows: MIC of the combination of the EOs/MIC of the EO alone. EOs were combined at MIC + MIC, MIC + 1/2 MIC, MIC + 1/4 MIC, MIC + 1/8 MIC, 1/2 MIC + 1/2 MIC, 1/2 MIC + 1/4 MIC, 1/2 MIC + 1/8 MIC, 1/4 MIC + 1/4 MIC, 1/4 MIC + 1/8 MIC, and 1/8 MIC + 1/8 MIC. The results were considered as synergy (FIC ≤ 0.5), addition (0.5 ≤ FIC ≥ 1), indifference (1 ≤ FIC ≥ 4), and antagonism (FIC > 4) [22].

### 4.9. Time–Kill Studies

The growth of all the test strains in contact with the corresponding antimicrobial, with *L. x intermedia* and *M. arvensis* EOs and with the different combinations of EO/EO and antimicrobial/EO was evaluated by calculating the change in the optical density of cells grown. In a 96-well sterile microplate, 90 μL of sterile nutrient broth and 10 μL of the strains were placed in each well from a stock, previously diluted to obtain a density of about 10^5^ CFU/mL. Antimicrobials and EOs were added at different concentrations for each well, depending on the results obtained during the evaluation of the MIC and of the fractional inhibitory concentration index. The microplate was incubated at 37 °C with an oscillating speed of 150 rpm, and the optical density (OD) was determined at 595 nm at predetermined time intervals (0, 6, 12 and 24 h) of exposure, using an automatic micro plate reader (Tecan Sunrise™). The experiments were replicated three times.

### 4.10. Anti-Biofilm Activity Determination

The effect of both EOs, by themselves or in combination (EO/EO and antimicrobial/EO) was tested on ’24 and 48 h old’ pre-formed biofilm, obtained using 96-well polystyrene microtiter plates, added with approximately 10^5^ CFU/mL of single microbial strains and incubated at 37 °C. After biofilm formation, the medium was gently aspirated and plates were washed three times with a sterile phosphate-buffered saline solution (PBS, pH 7.2) to remove planktonic bacteria, and the compounds were added at MIC concentration. Following an additional incubation for 24 h at 37 °C, the biofilm biomass was quantified according to the crystal violet staining method by Stepanovic et al. [56]. Absorbance values were measured at 570 nm using a microtiter plate reader.

### 4.11. Quantification of EOs Activity on Mature Biofilm by Fluorescence Assay Study

Wells containing mature ‘24 h old’ biofilm performed as above were washed with PBS solution, and the EOs, by themselves or in combination (EO/EO and antimicrobial/EO), were added at MIC concentration. After 24 h incubation treated and untreated wells (control) were washed twice with sterile PBS and stained by the “live/dead cells stain kit” (Thermo Fisher Scientific, Waltham, MA, USA), according to manufacturer instructions. The method is based on the use of propidium iodide (PI) as marker of dead cells and 5(6)-carboxyfluorescein diacetate (cFDA) to detect alive cells. After incubation in the dark at 37 °C for 30 min, the samples were washed twice with PBS and, to numerically quantify the amount of live and died cells, the fluorescence emission (CFDA excitation/emission: 485/528; PI excitation/emission: 528/645) was analyzed using a multi-well fluorescence plate reader (Synergy HTX, BIOTEK, Winooski, VT, USA). The results were expressed as relative fluorescence units (RFU).

### 4.12. Effectiveness of EOs Activity on Mature Biofilm by Fluorescence Microscopy Study

The effect of EOs on mature biofilm formation was evaluated in a 96-well microtiter plate, as described above. Subsequently, each well was washed two times with PBS to remove the unbound cells. Biofilm was fixed for 30 min with PBS-buffered 4% paraformaldehyde, then the samples were washed twice with PBS, treated with Prolong Gold antifade (PLGAR) (Thermo Fisher Scientific, Walthan, MA, USA) and stained by the “live/dead cells stain kit” (Thermo Fisher Scientific, Waltham, MA, USA), according to manufacturer instructions. The method is based on the use of propidium iodide (PI) as marker of dead cells and 5(6)-carboxyfluorescein diacetate (cFDA) to detect alive cells. After incubation in the dark at room temperature for 30 min, biofilm was visualized by epifluorescence microscopy Nikon Eclipse 90i imaging system, equipped with Normaski DIC optics (Nikon Instruments Inc., Melville, NY, USA). Samples were photographed with a DS-2Mv Nikon digital camera.

### 4.13. Statistical Analysis

Each experiment was replicated three times. The statistical significance was determined by *t*-test, Qui-square test, and ANOVA test. The *p*-values were considered to be significant at ≤0.05. 

## 5. Conclusions

In this study, *L. x intermedia* and *M. arvensis* EOs were selected from an initial panel of samples belonging to the *Lamiaceae* family on the basis of their preliminary activity against *S. agalactiae*, *C. albicans*, and lactobacilli, and their chemical composition was evaluated. The antimicrobial activity of the two EOs by themselves and in combination against all strains both in planktonic and in biofilm form was assessed. In particular, the association EO/EO and antimicrobial/EO gave interesting results. All of these findings represent an advantage in an attempt to overcome drug resistance emergency in a period like this, where even the most recent synthetic antimicrobial drugs are not enough effective against multidrug-resistant bacteria. 

In conclusion, these two EOs possess potential health benefit, especially that from *L. x intermedia*, and they could be valuable in the pharmaceutical fields, such as in gynecology and obstetrics, for the treatment of various symptoms and pathological conditions, and also for preventive purposes in detergents for personal hygiene, ointments, creams and ovules.

## Figures and Tables

**Table 1:** Qualitative and semi-quantitative analysis of *Lavandula x intermedia* and *Mentha arvensis* essential oils (EOs). Data are expressed as % relative peak area values ± standard deviation (SD).

Compound ^a^	*LRI* ^b^	*Lavandula x intermedia*	*Mentha arvensis*
α-Thujene	926	0.1 ^c^	-
α-Pinene	931	0.7 ^c^	0.7 ^c^
Camphene	946	0.5 ^c^	-
Sabinene	971	0.2 ^c^	0.3 ^c^
β-Pinene	974	0.5 ^c^	0.8 ^c^
β-Myrcene	990	1.0 ± 0.1	0.7 ^c^
*p*-Cymene	1024	0.3 ^c^	-
Limonene	1027	1.4 ^c^	3.6 ± 0.1
1,8-Cineole	1029	5.0 ^c^	0.2 ^c^
*cis*-Ocimene	1037	0.8 ^c^	-
*trans*-Ocimene	1047	0.9 ^c^	-
γ- Terpinene	1058	0.1 ^c^	-
Terpinolene	1087	0.4 ^c^	-
Linalool	1100	36.0 ± 0.1	0.1 ^c^
Camphor	1143	5.9 ± 0.1	-
Isopulegol	1146	-	0.8 ^c^
Menthone	1156	-	7.8 ± 0.1
Borneol	1165	4.0 ^c^	-
Isomenthone	1167	-	5.4 ^c^
Terpinen-4-ol	1177	2.7 ^c^	-
Menthol	1180	-	73.8 ± 0.2
α- Terpineol	1191	0.9 ^c^	0.1 ^c^
Pulegone	1245	-	0.5 ^c^
Piperitone	1259	-	0.5 ^c^
Linalyl acetate	1263	27.3 ± 0.2	-
Lavandulyl acetate	1293	1.8 ^c^	-
Menthyl Acetate	1297	-	2.1 ^c^
β-Bourbonene	1390	-	0.1 ^c^
β-Caryophyllene	1424	1.5 ^c^	0.3 ^c^
Germacrene D	1487	-	0.4 ^c^
Caryophyllene oxide	1592	0.2 ^c^	-
Total		92.1	98.2

^a^ Compounds are listed in order of elution. ^b^ Linear retention index (*LRI*) calculated on a HP-5 column. ^c^ SD < 0.05.

**Table 2:** Minimum inhibitory concentration (MIC) of EOs in comparison with the two reference antimicrobials (erythromycin and fluconazole). Data are expressed as µg/mL.

Strains	*Lavandula x intermedia*	*Mentha arvensis*	Erythromycin	Fluconazole
*Streptococcus agalactiae* 1	18	36	2	-
*Streptococcus agalactiae* 2	9	18	1	-
*Streptococcus agalactiae* ATCC 13813	18	18	0.125	-
*Candida albicans* 1	18	18	-	4
*Candida albicans* 2	9	144	-	4
*Candida albicans* ATCC 10231	18	72	-	0.25
*Lactobacillus* spp.1	144	9	0.5	1
*Lactobacillus* spp.2	144	18	0.25	1
*Lactobacillus* spp.3	72	18	0.25	1
*Lactobacillus* spp.4	72	18	1	0.5

## Supplementary Materials

The following are available online at https://www.mdpi.com/2079-6382/9/9/592/s1, Table S1: Agar Well Disk diffusion assay of EOs and common antimicrobial drugs. The inhibition diameters were measured in mm, Table S2: Minimal inhibitory concentration (MIC) and synergistic effect (FIC-INDEX) between EOs and antimicrobials (erythromycin and fluconazole). Data are expressed as µg/mL.
