# Supplementary material for: Phytochemical Composition and In Vitro Antimicrobial Activity of Essential Oils from the Lamiaceae Family against Streptococcus agalactiae and Candida albicans Biofilms
Source: Antibiotics (Basel). 2020 Sep 10;9(9):592. doi: 10.3390/antibiotics9090592 (PMC7558348; doi:10.3390/antibiotics9090592)
Supplement: Supplementary file 1 [file antibiotics-09-00592-s001.pdf]

Table S1. Agar Well Disk diffusion assay of EOs from *Lamiaceae* and reference antimicrobials.
The inhibition zones were measured in mm.

	Lavandula x intermedia	*Mentha arvensis*	*Origanum vulgare*	*Satureja montana*	*Thymus vulgaris*	Erythromycin	Fluconazole
Streptococcus agalactiae 1	27±1.1	22±2.0	30±1.5	40±1.2	50±1.0	32±1.4	-
Streptococcus agalactiae 2	35±1.9	24±1.4	30±1.00	35±1.3	40±1.7	35±2.0	-
Streptococcus agalactiae ATCC 13813	33±1.6	26±1.2	28±1.2	27±0.8	29±1.3	19±1.1	-
Candida albicans 1	28±1.2	25±1.4	19±1.5	16±0.6	20±0.4	-	30±1.3
Candida albicans 2	27±1.00	8±2.1	22±1.3	12±1.7	14±09	-	32±1.2
Candida albicans ATCC 10231	30±1.4	25±1.7	24±1.1	16±1.0	20±1.4	-	18±1.2
Lactobacillus spp.1	14±1.3	30±1.2	24±0.8	32±0.3	35±0.2	32±1.5	29±1.1
Lactobacillus spp.2	12±1.2	27±1.2	26±1.4	30±1.0	31±1.4	28±1.0	30±1.3
Lactobacillus spp.3	14±1.0	27±1.0	22±1.1	32±1.6	33±0.5	35±1.2	32±1.1
Lactobacillus spp.4	16±1.2	25±1.6	28±0.5	30±1.1	33±1.1	35±1.7	30±1.6

Table S2. Minimal Inhibitory Concentration (MIC) and synergistic effect (FIC-Index) between EOs and antimicrobials (erythromycin and fluconazole). Data are expressed as μg/mL.

	Essential oils, EOs (MIC)			Antibiotics (MIC)					
	Lavandula x intermedia	*Mentha arvensis*	FIC-INDEX of essential oils	Erythromycin	Fluconazole	FIC-INDEX *Lavandula x intermedia* and erythromycin	FIC-INDEX *Mentha arvensis* and erythromycin	FIC-INDEX *Lavandula x intermedia* and fluconazole	FIC-INDEX *Mentha arvensis* and fluconazole
Streptococcus agalactiae 1	18	36	0.16 (S)	2	-	0.31(S)	0.31(S)	-	-
Streptococcus agalactiae 2	9	18	0.31(S)	1	-	0.28(S)	0.31(S)	-	-
Streptococcus agalactiae ATCC 13813	18	18	0.125(S)	0.125	-	0.31(S)	0.31(S)	-	-
Candida albicans 1	18	18	0.31(S)	-	4	-	-	0.27(S)	0.31(S)
Candida albicans 2	9	144	0.13(S)	-	4	-	-	0.16(S)	0.27(S)
Candida albicans ATCC 10231	18	72	0.1(S)		0.25	-	-	0.14(S)	0.16(S)
Lactobacillus spp.1	144	9	0.27(S)	0.5	1	0.27(S)	0.37(S)	0.31(S)	0.4(S)
Lactobacillus spp.2	144	18	0.14(S)	0.25	1	0.27(S)	0.4(S)	0.27(S)	0.27(S)
Lactobacillus spp.3	72	18	0.16(S)	0.25	1	0.27(S)	0.31(S)	0.28(S)	0.27(S)
Lactobacillus spp.4	72	18	0.27(S)	1	0.5	0.31(S)	0.31(S)	0.28(S)	0.28(S)

(S): < 0.5 represents synergy

(I): > 0.5 < 4.0 represents indifference

(A): > 4.0 represents antagonism
